# High HTLV-1 Proviral Load Predates and Predicts HTLV-1-Associated Disease: Literature Review and the London Experience

**DOI:** 10.3390/pathogens13070553

**Published:** 2024-07-01

**Authors:** Graham P. Taylor, William Evans, Carolina Rosadas

**Affiliations:** 1 Section of Virology, Department of Infectious Disease, Faculty of Medicine, Imperial College London, London W2 1PG, UK; william.evans21@imperial.ac.uk (W.E.); c.rosadas-de-oliveira@imperial.ac.uk (C.R.); 2 National Centre for Human Retrovirology, St Mary’s Hospital, Imperial College Healthcare NHS Trust, London W2 1NY, UK

**Keywords:** HTLV-1, proviral load, diseases, HAM, PCR, outcome, prognostic marker

## Abstract

Human T cell lymphotropic virus type 1 (HTLV-1) is a retrovirus that infects lymphocytes and causes severe diseases. HTLV-1 proviral load (PVL), i.e., the number of host cells that carry HTLV-1 proviral DNA integrated into their genome, can be measured in peripheral blood mononuclear cells (PBMCs) using quantitative polymerase chain reaction. In this narrative review, we discuss the usefulness of HTLV-1 PVL quantification and share our experience acquired during more than 30 years of follow-up of people living with HTLV-1 in the UK. Patients with HTLV-1-associated myelopathy have higher PVL than those with asymptomatic infection. This is consistent across studies in different countries. High PVL predates symptom onset for both inflammatory and proliferative diseases. High PVL is essential but not sufficient for the development of HTLV-1-associated diseases. Therefore, PVL quantification can be used to support the care of people living with HTLV-1 by identifying those most at risk of HTLV-1-associated diseases.

## 1. Introduction

**Figure 1:**
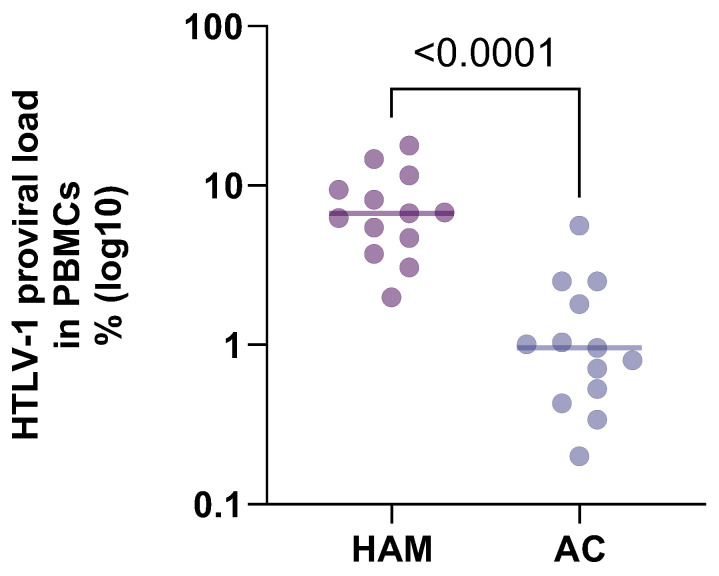
Comparison of HTLV-1 proviral load by disease state from Table 1. Proviral load in patients with HTLV-associated myelopathy is compared to patients with asymptomatic HTLV-1 infection.

Human T cell lymphotropic virus type 1 (HTLV-1) is a retrovirus with global distribution. HTLV-1 is transmitted by sex, vertically, and by contact with infected blood or tissues. There is a strong body of evidence that links HTLV-1 with a range of diseases and shows that, at the time of diagnosis of disease, HTLV-1 proviral load (PVL) in peripheral blood mononuclear cells (PBMCs) is high (HTLV-associated myelopathy (HAM) [1,2,3,4,5,6], Adult T cell leukaemia/lymphoma (ATL) [3], HTLV-1-associated uveitis [7], kerato-conjunctivitis sicca [8] and infective dermatitis [9,10,11]). Despite differences in methods, standards and patients, the results for proviral load in patients with HAM compared to asymptomatic carriers are strikingly similar (see Table 1 and Figure 1) [1,3,4,12,13,14,15,16,17,18,19,20,21], with a proviral load on average 7 times higher in patients with HAM than in asymptomatic carriers across studies in different populations spanning 25 years. The question therefore is whether the disease caused the high proviral load to occur or whether the high proviral load predated and thus predicts the disease. In this narrative review, we will discuss the current knowledge on HTLV-1 proviral load, focusing on its association with disease, and share our experience from a National HTLV Clinical Service in London.

## 2. The London Experience

In 1992, we established a clinic in London to provide care for patients diagnosed with HTLV-1 and to better understand the natural history of this infection. We were aware of the already established associations of HTLV-1 with ATL and with a number of conditions characterised by inflammation, such as HAM, uveitis, myopathy and thyroiditis. We are still learning regarding the overarching question, namely, what is the full impact and spectrum of diseases caused by HTLV-1 infection?

A second and more focused question was the following: What is the importance of the viral burden? To this end, we established HTLV-1 proviral load quantification, first by limiting dilution and nested PCR and from 2000 by real-time quantitative PCR. Since 2000, as a matter of clinical routine, proviral load is measured in all patients at all visits. HTLV-1 proviral load results are reported as HTLV-1 DNA copies/100 PBMCs, which we abbreviate to %.

In 1999, we reported on our first 20 initially asymptomatic carriers [22]. The range of proviral loads was wide but stable, and high proviral load predated the onset of HTLV-1-associated myelopathy and uveitis, seen in one patient each. By 2013, we had accumulated data on more than 400 patients attending the clinic. Patients who were asymptomatic (n = 211) had a median proviral load (using our assay) of 1.8%, whilst patients with HAM (n = 84) had a median proviral load of 14.7%, and none had a proviral load less than 1.7% [3]. As expected, patients with leukaemia due to ATL had even higher proviral loads (50%), whilst even patients with ATL-lymphoma and no evidence of involvement of the peripheral blood had high median proviral load (7.9%). Through examining sequential, prospectively acquired data, we observed that in asymptomatic carriers with a minimum of 4 years (median 6.5 years) follow-up, proviral load decreased from 1.7% to 1%. In 2013, we reported on incident ATL within the cohort. Of 153 initially asymptomatic carriers followed up for a median of 4.5 years, 4 developed incident ATL and all had a pre-onset of disease proviral load > 10% [23]. By 2020, out of a total cohort of 658 people living with HTLV-1, 6 had developed ATL, with documented high proviral load predating the onset of ATL 2–10 years earlier [24]. On the basis of these results, which indicated that HTLV-1-associated disease was not occurring in asymptomatic carriers with proviral load < 1% and that proviral load was stable over many years (and tending to decrease rather than increase), we reduced the frequency of routine clinic reviews for these patients with low (<1%) proviral load to annual. Furthermore, anecdotal data from two blood donors who had become infected between donations suggested that proviral load plateaued in the first months following primary infection. This was confirmed in a prospective study of three transplant recipients who inadvertently received organs from a single donor prior to diagnosis of HTLV-1 infection in the donor (who was perceived to be HTLV low risk) [25]. Proviral load increased rapidly during early infection, at its maximum doubling every day before plateauing by day 45 from infection and thereafter remaining stable.

Having identified high proviral load as a risk for HTLV-1-associated disease, we have more recently identified additional markers that predict the development of HAM and ATL, namely activated T cells [24] and the clonal expansion of HTLV-1 infected T cells [25], respectively. In both cases, having a proviral load > 1.8% (i.e., the median proviral load among asymptomatic carriers) is the baseline requirement. Thus, in our clinic, we have only observed incident HAM in patients with a “HAM-like” viral-immune phenotype, which comprises having a proviral load > 2.1% in addition to a high expression of T cell activation markers (*p* = 0.004 comparing incidence to non-HAM-like asymptomatic carriers) [26]. Likewise, incident ATL has only been observed in asymptomatic carriers with both a proviral load > 4% and a high oligoclonality score on flow cytometry [27].

Thus, our data altogether confirmed that low proviral load has a good predictive value for asymptomatic infection, whilst high proviral load identifies those for additional risk evaluation. These observations have enabled much more targeted use of clinical resources, reduced the attendances of low-risk (low proviral load) asymptomatic carriers and opened the door to early interventions.

## 3. HTLV-1 Proviral Load Results Globally

How does our experience that high proviral load predates and predicts disease compare with the literature? Using search terms for HTLV-1, cohort, incident, HAM and ATL in Pubmed, the following papers were identified.

In a Brazilian cohort in Minas Gerais State, HTLV-1 proviral load was six-fold higher in patients with HAM, a median of 3.36%, compared with the asymptomatic patients who had a median of 0.48% (*p* < 0.001). Furthermore, having an HTLV-1 proviral load > 1.14% correctly identified 78% of the patients with HAM [28]. More severe disease, e.g., progression to wheelchair dependency was associated with higher proviral load. Although there were no incident cases in this small cohort of 75 asymptomatic carriers to confirm that high proviral load predates and thus predicts the development of HAM, HTLV-1 proviral load was found to be stable in both patient groups, especially in those with high proviral load, in agreement with our experience [3].

In the Minas Gerais HTLV cohort, HTLV-1 proviral load was stable across an average of 10 years for asymptomatic carriers, was higher in patients with HAM (baseline median PVL 5.56%) than in asymptomatic carriers (1.02%) and was greater than 1% in each of the 6 (out of 88) initially asymptomatic carriers who developed HAM during follow-up. The authors note that proviral load did not increase in these six patients prior to or at the time of onset of HAM but was stable [19].

During 8 years of follow-up of a longitudinal cohort in Salvador, Bahia, Brazil, incident neurological manifestations were observed in 30% of the cohort, including incident HAM in 4 of the 251 participants. High HTLV-1 proviral load (>5%) predated disease [29].

In a distinct Brazilian cohort from São Paulo, 175 apparently asymptomatic participants underwent detailed neurological evaluation. Forty-two (24%) were found to have a constellation of three or more of the following: bladder symptoms, neurological symptoms or signs, skin lesions, oral disease or visual disturbance suggestive of early HTLV-1-associated inflammation. This condition was termed intermediate syndrome (IS) and was associated with high HTLV-1 proviral load (mean 1.7%) compared to those without IS (mean 0.25% *p* = 0.007) [30].

Children in Brazil with infective dermatitis have a high proviral load, a median of 11.1% compared with asymptomatic children (median 1.2%), and are at high risk of developing HAM: 7/18 of these children developed HAM during 4 years of follow-up, with high proviral load preceding the onset of HAM [11].

In a prospective study of HTLV-1-infected blood donors in the USA, HTLV-1 proviral load was 10-fold higher in those with HAM than those who did not have HAM [31]. Incident HAM was observed during follow-up in two females with baseline proviral loads of 8.4 and 1.6%.

One of the earliest HTLV-1 cohorts was established in Miyazaki in Kyushu, southwest Japan. Incident ATL occurred in four participants, with an earliest sample 3–8 years before the manifestation of ATL. Their median proviral load was 4.9% [32]. This was significantly higher (*p* = 0.03) than in 37 age-matched controls (0.8%). Further evidence of the predictive power of HTLV-1 proviral load also comes from Japan, where, in a nationwide prospective study of asymptomatic carriers recruited between 2002 and 2008, incident ATL occurred in 14/1218 participants. The median proviral load in this cohort at baseline was 1.6%, with 25% of study participants having a proviral load > 4.54%. The median baseline proviral load in those in whom ATL developed was 10.3% (range 4.2–28.6%) [33]. This finding that ATL occurs on a background of high (>4%) proviral load was also observed in the UK cohort, which was predominantly of African ancestry. This cut-off is now incorporated in the clinical protocol at the National Centre for Human Retrovirology, London, UK, to determine which patients are monitored more frequently for ATL and investigated for pre-ATL.

There is thus evidence from several prospective studies in Asia, the Americas and Europe that high HTLV-1 proviral load is not the consequence of the development of HTLV-1-associated disease but predates and predicts HTLV-1-associated disease and that this is seen not only for ATL but also for HAM. What about other HTLV-1-associated inflammatory disease?

The link between HTLV-1 and pulmonary disease was initially reported from Japan in 1989 as a finding in patients with HAM [34] and patients with HTLV-1-associated uveitis [35]. Okada et al. reported high rates (30%) of pulmonary disease using CT among 320 patients with HTLV-1 in 2006 [36], including 50 patients with bronchiectasis. In patients with bronchiectasis presenting to Alice Springs Hospital, 72% were HTLV-1 seropositive [37]. The mortality rate from bronchiectasis is high (34.2%) and occurs in early life (median age at death 42.5 years) [38]. HTLV-1 was associated with higher rates of cor pulmonale and disease-specific mortality [38]. Furthermore, HTLV-1 proviral load was 100-fold higher among patients with bronchiectasis than case controls, and high proviral load correlated with pulmonary disease severity [39]. In the UK, HTLV-1 is also associated with bronchiectasis and is particularly common among patients with HAM [40]. Overall, bronchiectasis was found in 3.4% of the HTLV-1 cohort, with a relative risk of 8.4 for patients already diagnosed with HAM. HTLV-1 is acknowledged as a cause of bronchiectasis by the British Thoracic Society [41]. A similar association between HAM and lung disease including bronchiectasis was reported from a prospective study of people living with HTLV-1 in the eastern Amazon, Brazil. None had clinical evidence of pulmonary disease prior to enrolment, yet 73.5% of the patients with HAM and 22% of those without HAM had evidence of lung disease on high-resolution CT images, with bronchiectasis/bronchiolectasis being the most common finding, observed in 40% of patients with HAM [42]. These findings support the suggestion that bronchiectasis is a consequence of HTLV-1-associated inflammation.

High HTLV-1 proviral load among indigenous Australians has also been associated with bloodstream infections [43], urinary infections [44], and most recently with common non-communicable diseases including kidney disease and diabetes [45]. In an Iranian cohort, high HTLV-1 proviral load (>5%) was associated with an increased risk of having diffuse coronary artery disease (OR = 6.87, 95%CI = 1.34–35.05%, *p* = 0.016) [46].

## 4. Final Considerations

High HTLV-1 proviral load has been found to be associated with a broad range of HTLV-1-associated diseases found across the world. Data from six cohorts totalling 19 incident cases of HAM among initially asymptomatic carriers and 20 incident cases of ATL among people living with HTLV further demonstrate that high proviral load predates both inflammatory (HAM) and malignant (ATL) HTLV-1-associated disease. In the London clinic, using qPCR the median proviral load among asymptomatic carriers is 1.8%, and HTLV-1-associated disease is only observed in PLWH above 1% (and above 4% for ATL). Although there is broad agreement that HTLV-1 proviral load is significantly (approaching 10-fold) higher in those who have or who will develop HTLV-1-associated disease, each centre should determine the appropriate cut-off according to their methods and assay. However, it is likely that this will be in the region of a proviral load of 1%. This will allow PLWH at a lower proviral load to be reassured and followed up less frequently. In our practice, we continue to offer annual review of PLWH and low viral load, as our data are still only based on a maximum of three decades of follow-up. Although there have been reports of proviral load increasing prior to the onset of disease, we have not observed this amongst >500 asymptomatic carriers, but offering annual or even biennial review addresses this possibility.

Although the predominant impact of HTLV-1 among Indigenous Australians appears to differ from other geographic regions (noting the potential impact of multiple differences in virus strain, host, environment and time of infection), there is a clear overlap with pulmonary disease associations, particularly bronchiectasis, which in addition to Australia has been reported in association with HTLV-1 in South America, Japan and Europe. Closing the data gap by demonstrating that high proviral load also predates the onset of HTLV-1-associated non-communicable diseases [45] would be useful to better understand this relationship but requires adequate follow-up of initially asymptomatic carriers and should include other measures such as markers of inflammation. More research on the impact of HTLV-1 proviral load on the risk of transmission would also be beneficial. Meanwhile, since high proviral load is essential but not sufficient for the development of HTLV-1-associated diseases, proviral load monitoring can be used to support the care of people living with HTLV-1 by identifying those most at risk of HTLV-1-associated diseases. The use of the quantification of infection burden to determine management is increasingly common in virology, and in HTLV infection a single measurement might allow the targeted utilisation of resources.

## Figures and Tables

**Table 1:** Comparison of median HTLV-1 proviral load in patients with HTLV-1-associated myelopathy (HAM) compared with asymptomatic carriers (ACs) from published studies.

First Author	Year	Median Proviral Load HAM (/10^2^ PBMC)	n	Median Proviral Load AC (/10^2^ PBMC)	n	PVL HAM/AC Ratio	Population	Reference
Nagai, M.	1998	5.44	202	0.34	200	16	Japanese	[1]
Dehee, A.	2002	9.39	5	0.43	5	21.8	Afro-Caribbean	[4]
Olindo, S.	2005	8.13	100	0.8	34	10.2	Afro-Caribbean	[21]
Yakova, M.	2005	6.24	4	1.01	8	6.2	Afro-Caribbean	[17]
Best, I. et al.	2006	17.79	35	5.61	33	3.2	Peruvian	[16]
Grassi, M.F.R. et al.	2011	11.6	47	0.71	189	16.3	Brazilian	[12]
Demontis, M.A.	2012	14.7	84	1.8	211	8.2	UK (Afro-Caribbean/Caucasian)	[3]
Martins, M.L. et al.	2017	3.73	46	0.96	351	3.9	Brazilian	[19]
Tarokhian, H. et al.	2017	3.06	33	2.5	38	1.2	Iranian	[15]
Domingos, J.A. et al.	2017	6.71	11	2.51	29	2.7	Brazilian	[18]
Rajaei, T. et al.	2019	4.69	22	0.53	22	8.8	Iranian	[20]
de-Mendoza et al.	2023	6.77	58	1.04	393	6.5	Spanish	[13]
Manzarinejad, M. et al.	2023	1.99	30	0.2	30	4.8	Iranian	[14]

## Data Availability

The data presented in this study are available in the manuscript.
